# Impact of Soil Warming on the Plant Metabolome of Icelandic Grasslands

**DOI:** 10.3390/metabo7030044

**Published:** 2017-08-23

**Authors:** Albert Gargallo-Garriga, Marta Ayala-Roque, Jordi Sardans, Mireia Bartrons, Victor Granda, Bjarni D. Sigurdsson, Niki I. W. Leblans, Michal Oravec, Otmar Urban, Ivan A. Janssens, Josep Peñuelas

**Affiliations:** 1 Consejo Superior de Investigaciones Científicas (CSIC), Global Ecology Unit CREAF-CSIC-UAB, 08193 Bellaterra, Spain; j.sardans@creaf.uab.cat (J.S.); mireiabartrons@gmail.com (M.B.); victorgrandagarcia@gmail.com (V.G.) Josep.Penuelas@uab.cat (J.P.); 2 Ecological and Forestry Applications Research Centre, 08193 Cerdanyola del Vallès, Spain; 3 BETA Technological Centre (Tecnio), Aquatic Ecology Group, University of Vic-Central University of Catalonia, Vic, 08500 Barcelona, Spain; 4 Agricultural University of Iceland, IS-311 Borgarnes, Iceland; bjarni@lbhi.is (B.D.S.); niki.leblans@uantwerpen.be (N.I.W.L.); 5 Department of Biology, University of Antwerp, BE-2610 Antwerp, Belgium; ivan.janssens@uantwerpen.be; 6 Global Change Research Institute, The Czech Academy of Sciences, Belidla 986/4a, CZ-60300 Brno, Czech Republic; oravec.m@czechglobe.cz (M.O.); urban.o@czechglobe.cz (O.U.)

**Keywords:** climate change, warming, geothermal bedrock channels, grassland, metabolome, Iceland

## Abstract

Climate change is stronger at high than at temperate and tropical latitudes. The natural geothermal conditions in southern Iceland provide an opportunity to study the impact of warming on plants, because of the geothermal bedrock channels that induce stable gradients of soil temperature. We studied two valleys, one where such gradients have been present for centuries (long-term treatment), and another where new gradients were created in 2008 after a shallow crustal earthquake (short-term treatment). We studied the impact of soil warming (0 to +15 °C) on the foliar metabolomes of two common plant species of high northern latitudes: *Agrostis capillaris*, a monocotyledon grass; and *Ranunculus acris*, a dicotyledonous herb, and evaluated the dependence of shifts in their metabolomes on the length of the warming treatment. The two species responded differently to warming, depending on the length of exposure. The grass metabolome clearly shifted at the site of long-term warming, but the herb metabolome did not. The main up-regulated compounds at the highest temperatures at the long-term site were saccharides and amino acids, both involved in heat-shock metabolic pathways. Moreover, some secondary metabolites, such as phenolic acids and terpenes, associated with a wide array of stresses, were also up-regulated. Most current climatic models predict an increase in annual average temperature between 2–8 °C over land masses in the Arctic towards the end of this century. The metabolomes of *A. capillaris* and *R. acris* shifted abruptly and nonlinearly to soil warming >5 °C above the control temperature for the coming decades. These results thus suggest that a slight warming increase may not imply substantial changes in plant function, but if the temperature rises more than 5 °C, warming may end up triggering metabolic pathways associated with heat stress in some plant species currently dominant in this region.

## 1. Introduction

The global mean surface temperature at the end of the century (2081–2100) is likely to increase between 2.6 and 4.8 °C, and even more towards the poles (2–8 °C) [1]. The impact of climate change [2] on terrestrial plants is difficult to predict because it depends on many direct effects, e.g., on temperature and water availability, but also indirect effects, e.g., on nutrient availability or biotic relationships [3,4,5]. The effect of warming should be species-specific and dependent on the range of temperatures to which each species is adapted to live in, and of the growth strategy of each species. Stress-tolerant species, for instance, have lower plasticity capacity than competitor or ruderal species to adapt to environmental changes [6]. Moreover, there are species able to live in a wide range of temperatures, whereas others can only live in a narrow range. Northern ecosystems are characterised by stress-tolerant species (S) [7], adapted to nutrient-poor conditions, and having low foliar nitrogen and phosphorus concentrations [8,9]. The species resistant to stress usually have low phenotypical plasticity [7]*.* This low phenotypical plasticity can even be a great constrain, because the capacity of organisms to increase their activity due to global warming could be limited by several variables, such as water and nutrient availabilities [10,11,12]. This is especially relevant in sub-arctic ecosystems, where warming largely impacts on soil nutrient availability, plant production, and reproduction [13,14,15,16]. Recent meta-analyses of individual plant responses to moderate warming have showed that the species capacity to respond depends on their growth form [17,18]. Moreover, extreme climatic events and/or fast climate change can reach a critical point for some species by surpassing the thresholds of resistance to the new conditions [19,20,21,22,23,24,25].The analysis of the subarctic plants of Iceland will thus be especially useful for understanding the sensitivity of plant metabolomes to warming, and the plant capacity to cope with warming in high latitudes.

Ecometabolomics is a powerful tool that can assess the effects of the ecological organism–environment interactions, by detecting the final phenotypic response of an organism, identifying the metabolic pathways that are up- and down-regulated in response to environmental changes, and identifying the metabolites and metabolic pathways responsible for stress resistance in plants [26,27,28]. Ecometabolomics has recently been used to monitor the phenotypic changes in response to shifts in temperature [28,29,30,31,32,33,34,35,36], observing that heat stress increases the concentrations of saturated fatty acids [37,38], which increase the thermotolerance of thylakoid [39] and plasma [40] membranes. Increases in amino acids concentrations have been also observed [41,42], consistent with the accumulation of heat-shock proteins, which are responsible for stress resistance in heat-exposed conditions [43,44]. However, little is known about the effects of warming on the metabolomes of subarctic grassland plant species, despite that they suffer more intense warming than many other biomes. 

We studied soil short-term and long-term thermal gradients in Iceland. They have a combination of wide temperature gradients and different exposure times, that allowed us to simultaneously search for tipping points in the responses to soil warming, and distinguish between evolutionary adaptation, i.e., long-term responses, and immediate, short-term responses to warming. We investigated the effects of short- and long-term warming on the foliar metabolomes of the grass *Agrostis capillaris* and the herbaceous dicotyledon *Ranunculus acris*. *Agrostis capillaris* is one of the most common grass species in Eurasia, including the North Atlantic Islands. It is a stoloniferous perennial grass that forms dense swards of fine leaves growing on neutral and acid soils [45]. *Ranunculus acris* is a perennial dicot forb that grows across Europe and temperate areas of Asia [46]. In America and Oceania, it is a successful invasive species. We tested the hypotheses that (1) changes in metabolome are species-specific, and (2) these changes are further modulated by the length of the warming period. The large gradients of soil temperature in these valleys of southern Iceland allow the study of the impact of the predicted temperature rise at short and long terms, and of the likely abrupt shifts in the progressive soil warming (to +15 °C). The site of long-term warming has likely been warmed for centuries, or for at least 50 years, and the site of short-term warming has been warmed only since 2008.

## 2. Material and Methods

### 2.1. Description of the Study Area

#### Study Sites

**Figure 1:**
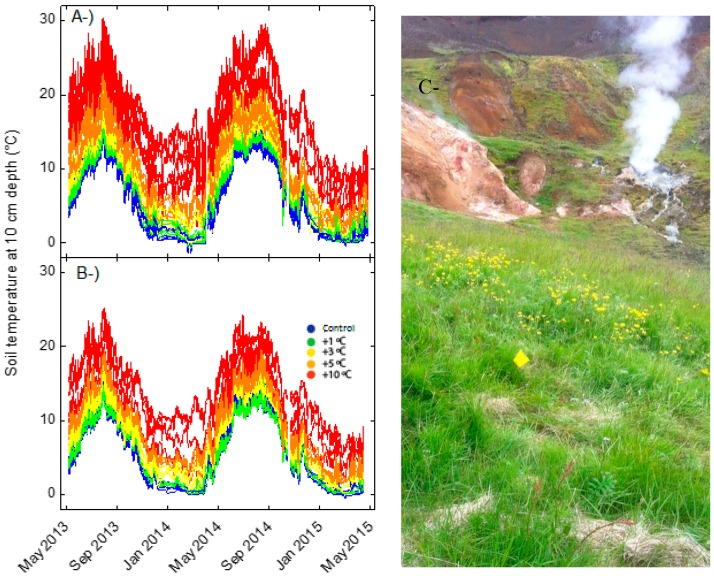
Soil temperatures at a depth of 10 cm from May 2013 to May 2015 in each measurement plot at the sites of (**A**) short-term warming site and (**B**) long-term warming site. (**C**) Natural soil warming in a natural grassland in Iceland; the yellow flowers are *Ranunculus acris*.

The two grasslands are located in the southern part of Iceland, near the town of Hveragerði (64°00′01′′ N, 21°11′09′′ W; 100–225 m a.s.l.). The mean annual temperature is 5.2 °C, with means of −0.1 °C in the coldest month (January), and 12.2 °C in the warmest (July), and the mean annual precipitation is 1457 mm (Icelandic Met Office, Reykjavík, Iceland, 2016). The soil is a silandic andosol (FAO WBR), also known as brown andosol, of volcanic origin. This type of soil drains freely, is rich in allophanic clay minerals and ferrihydrates, and has evolved from aeolian and tephral materials originating from neighbouring active volcanos [47,48]. The geothermal activity in this area, mainly in the form of hot springs and fumaroles, originates from the Hengill volcanic system. This system is at the intersection of three volcanic zones: the Hengill, Hrómundartindur, and Hveragerði zones [49,50]. The temperature of soil is variable throughout the year, and the differences of temperature of the studied sites with respect normal temperature in soils (controls) is maintained throughout the year (Figure 1a). 

The site of long-term warming is in a valley <3 km northwest of Hveragerði, and has been known for its geothermal activity for centuries [51]. Transects in this area were used to study the long-term effects of soil warming. The site of short-term warming is in a valley that has only been warmed since an earthquake in May 2008 modified the channels of hot water within the bedrock [52]. Transects of this grassland are situated near the campus of Hveragerði University, and were used to study the short-term effects of soil warming on plant metabolomics.

The selected plants were a monocotyledonous grass species (*A. capillaris*) and a dicotyledonous herbaceous species (*R. acris*), both of which are dominant or sub-dominant species in the types of grassland at the study sites [53]. A PCoA of the total species composition shows that (1) the control plots of the short- and the long-term warmed grassland have a very similar species composition, and (2) that there are no major changes in dominant species along the soil warming gradients, neither in the long-term warming site nor in the short-term warming site (data not shown). 

### 2.2. Experimental Design

Five replicate transects were established along temperature gradients in the two valleys. Each transect consisted of six levels of soil warming. The orientations of the slopes were as similar as possible for all transects at both sites. 

The temperature levels were originally established in 2013 by collecting point measurements within 2 × 2 m permanent study plots, where the soil temperature at a depth of 10 cm was monitored hourly at one location in each plot (Figure 1) by automated temperature loggers (HOBO TidbiT v2, Onset Computer Corporation, Manhattanm, NY, USA). Each temperature level represented the original spatial average soil temperature of each plot. Six temperature levels were tested: control, +1 °C, +3 °C, +5 °C, +10 °C, and +15 °C. 

On the 29 May 2008, a major earthquake (magnitude 6.3 on the Richter scale) occurred in S Iceland (Halldorsson & Sigbjörnsson 2009). The earthquake affected geothermal systems and increased the temperature in the soil above by radiative heating in the new geothermal bedrock channels [54]. The recently warmed area is covered by unmanaged treeless grasslands dominated by *Agrostis capillaris* grass, some herbs and moss, hereafter termed “SW” (shortly warmed site). The soil type at both sites is silandic andosols (IUSS Working Group WBR 2015: a volcanic soil type, also known as brown andosol [55].

The second study site “LW” (long-term warmed site) is located 2.0–2.5 km NW of SW on older geothermal Ts gradients, in Grændalur. It is covered by the same grassland type as SW and on the same soil type. There, the earliest survey of geothermal hot spots was made in 1963–1965 (45 years prior to the 2008 earthquake; Kristján Sæmundsson, personal communication). The geothermal activity has most likely been persistent in Grændalur (Green valley) for centuries, as according to local knowledge, its name comes from the fact that the subarctic grasslands on the warmest hot spots remain green during early winter, and turn green after the worst of winter has passed. Additional evidence for persistent geothermal warming at LW includes the geothermal clay layers found at various depths in the subsoil profile, thus indicating that over longer time periods, the warming may have fluctuated somewhat, as was observed at other nearby hot spots following the 2008 earthquake (Daebeler et al., 2014). The altitudes were the same in the two sites: 100–225 m a.s.l. Further information on the study sites and the experimental design are provided by Sigurdsson et al., 2016. 

### 2.3. Collection and Preparation of Tissue Samples 

Samples were collected in the middle of the growing season in July 2015; 113 foliar samples were collected (2 species × 2 sites (short- and long-term warming) × 6 warming treatments × 5 transects (plots)). The procedure for sample preparation is described in detail by Rivas-Ubach et al., 2013. Briefly, frozen samples were lyophilised and stored in plastic cans at −80 °C. The samples were ground with a ball mill (Mikrodismembrator-U, B. Braun Biotech International, Melsungen, Germany) at 1700 rpm for 2 min, producing a fine powder that was stored at −80 °C until the extraction of the metabolites. See the supplementary material of Gargallo-Garriga et al., 2014 for details.

### 2.4. Extraction of Metabolites for Analysis by Liquid Chromatography-Mass Spectrometry (LC-MS)

The extraction of metabolites followed the protocol of t’Kindt et al., 2008 with minor modifications. The Eppendorf tubes contained 150 mg of powder from each sample, and 1 mL of MeOH–H_2_O (80:20) was then added to each tube. The tubes were vortexed for 15 s, sonicated for 2 min at room temperature, and then centrifuged at 1100 g for 15 min. After centrifugation, 0.7 mL of the supernatant from each tube was collected using crystal syringes, filtered through 0.22 µm pore microfilters, and transferred to a labelled set of high-performance liquid chromatography (HPLC) vials. The vials were stored at −80 °C until the LC-MS analysis. This procedure was repeated, for two extractions of the same sample. 

### 2.5. LC-MS Analysis

LC-MS chromatograms were obtained with an UltiMate 3000 HPLC system (Thermo Fisher Scientific, Waltham, MA, USA) coupled to an LTQ Orbitrap XL high-resolution mass spectrometer (Thermo Fisher Scientific, San Jose, CA, USA) equipped with an HESI II (heated electrospray ionisation) source. Chromatography was performed on a reversed-phase C18 Hypersil gold column (150 × 2.1 mm, 3 µm particle size; Thermo Scientific, Waltham, MA, USA) at 30 °C. The mobile phases consisted of acetonitrile (A) and water (0.1% acetic acid) (B). Both mobile phases were filtered and degassed for 10 min in an ultrasonic bath prior to use. The elution gradient, at a flow rate of 0.3 mL min^−1^, began at 10% A (90% B) and was maintained for 5 min. The elution was then gradually changed to 10% B (90% A) during the next 15 min and maintained for 5 min, to flush the system. The initial proportions (10% A and 90% B) were subsequently recovered, and the column was then washed and stabilised for 5 min before the next sample was injected. The injection volume of the samples was 5 µL. HESI was used for MS detection. All samples were injected twice, once with the ESI operating in negative ionisation mode (−H), and once in positive ionisation mode (+H). The Orbitrap mass spectrometer was operated in FTMS (Fourier Transform Mass Spectrometry) full-scan mode with a mass range of 50–1000 *m*/*z* and high-mass resolution (60, 000 Da). The resolution and sensitivity of the spectrometer were monitored by injecting a standard of caffeine after every 10 samples, and the resolution was further monitored with lock masses (phthalates). Blank samples were also analysed during the sequence. The assignment of the metabolites was based on the standards, with the retention time and mass of the assigned metabolites in both positive and negative ionisation modes.

### 2.6. Processing of LC-MS Data 

The LC-MS raw data files were processed using MZmine 2.10 [56] (see Table S3 of the Supporting Information for details). The chromatograms were base-line-corrected, deconvoluted, aligned, and filtered, and the numerical database was exported in “csv” format. Metabolites were assigned by comparison with the analysed standards (retention time and mass spectrometry). Assigned variables corresponding to the same molecular compounds were summed. The LC-MS data for the statistical analyses corresponded to the absolute peak area at each retention time (RT). The area of a peak is directly proportional to the amount of its corresponding (assigned) metabolite in the sample. A change in the area of a peak thus indicates a change in the amount of its assigned metabolite. 

### 2.7. RNA:DNA Ratio

Quantification of nucleic acids followed Bentle et al., Wagner et al. and Gorokhova et al. [57,58,59] methods with numerous modifications. Reagents used were RNA (baker’s yeast *S. cerevisiae*, Sigma-Aldrich, Saint Louis, MO, USA) and DNA (calf thymus, Sigma-Aldrich, Saint Louis, MO, USA), RNase, DNase-free (Sigma-Aldrich, Saint Louis, MO, USA), *N*-lauroylsarcosine sodium salt (Sigma-Aldrich, Saint Louis, MO, USA), UltraPure^™^ DNase/RNase-Free Distilled Water (Life Technologies, Carlsbad, CA, USA), RiboGreen^™^ RNA Quantitation Kit (Molecular Probes, Inc., Eugene, OR, USA), PicoGreen^™^ dsDNA Quantitation Reagent (Molecular Probes, Inc., Eugene, OR, USA), TE buffer (RNase-free from RiboGreen^™^ RNA Quantitation Kit, Molecular Probes, Inc., Eugene, OR, USA), extraction buffer (1% *w*/*v N*-lauroylsarcosine sodium salt in TE buffer), and standard buffer (0.2% *N*-lauroylsarcosine sodium salt in TE buffer). 

Dry leaf tissue (5 mg) and dry soil (150 mg) frozen at −80°C were homogenised into powder (in liquid nitrogen). Samples were diluted in standard buffer and shaken at room temperature on a multiple vial head for 1.5 h, then diluted 1:4 with TE buffer, and shaken for an additional 15 min. Negative controls containing all chemicals, but no sample, were included in every set of samples and processed in the same way. Samples were analysed immediately using a Multilabel Plater Reader (VICTOR3, Perkin Elmer, Waltham, USA, filters: 485 nm for excitation and 535 nm for emission) and black solid flat-bottom microplates (Perkin Elmer, Waltham, MA, USA). Staining with Ribogreen, followed by RNase digestion, was used to estimate RNA by calculating the difference between initial and remaining sample fluorescence. From this difference, RNA concentrations in the samples were calculated based on the RiboGreen standard curve against known RNA concentrations. DNA was measured with PicoGreen, and DNA concentrations were calculated according to the respective DNA standard curves. Recoveries were determined by spiking 10 subsamples (five for RNA and five for DNA) of soil and leaves. The final yields of internal standard RNA and DNA were 67 ± 7% and 91 ± 4%, respectively.

### 2.8. Statistical Analyses 

The HPLC-MS data were analysed by univariate and multivariate statistical analyses. The method used for data pretreatment was auto scaling, also called unit variance scaling, that is commonly applied and uses the standard deviation as the scaling factor [60]. We conducted permutational multivariate analyses of variance (PERMANOVAs) [61] using the Euclidean distance, with site (short- and long-term warming), species (*A. capillaris* and *R. acris*), warming treatment (six levels of warming), and plant, as fixed factors, and plots as random factors. PERMANOVA is based on multiple random permutations of dependent variables that allow to build a random distribution of all the variables at once. This determines whether the experimental output distribution is random (null hypothesis) or not (alternative hypothesis), and thus, whether the independent factors (in our case site, species and temperature levels) have significant effects on overall variables (metabolites distribution). Multivariate ordination principal component analyses (PCAs) (based on correlations), the solid validation procedures (Figure S1) and partial least squares discriminant analyses (PLS-DAs) were also performed to detect patterns of sample ordination in the metabolomic. The profiles of leaves from different sites were additionally submitted to separate PCAs. The PC scores of the cases were subjected to one-way ANOVAs, to determine the statistical differences amongst groups with different levels of the categorical independent variables (species, site, and warming treatment). We additionally conducted ANOVA-simultaneous component analyses (ASCA) [62] to separate the variation in the total dataset by the different warming treatments in each species and site. The PERMANOVAs, PCAs, PLS analyses, and generation of clustered image maps were conducted by the mixOmics package of R software (R Development Core Team 2015). The ASCA were conducted by the lmdme package of R software (R Development Core Team 2015). The Kolmogorov–Smirnov (KS) test was performed on each variable to test for normality. All assigned and identified metabolites were normally distributed, and any unidentified metabolomic variable that was not normally distributed was removed from the data set. Statistica v8.0 (StatSoft, Tulsa, OK, USA) was used for the ANOVAs, post hoc tests, and KS tests.

## 3. Results

### 3.1. Differences between Species, Sites and Warming Levels

The metabolomes of *A. capillaris* and *R. acris* differed (PERMANOVA pseudo-*F* = 243; *p* < 0.001). The metabolomes also differed significantly between the SW and LW sites (pseudo-*F* = 13.2; *p* < 0.001) and were marginally responsive to warming (pseudo-*F* = 2.02; *p* < 0.1). Interactions were significant between species and site (pseudo-*F* = 6.29; *p* < 0.001) and between site and warming (pseudo-*F* = 2.03; *p* < 0.05), but not between species and warming (pseudo-*F* = 1.26; *p* > 0.05). The overall metabolomic profiles of both species shifted abruptly and nonlinearly between 5 and 10 °C above the control temperature (Table S1).

Soil pH and C and N concentrations differed between sites (pH: *F* = 5.42, *p* < 0.05; C: *F* = 10.5, *p* < 0.001; N: *F* = 155, *p* < 0.001) and amongst warming levels (pH: *F* = 6.03, *p* < 0.001; C: *F* = 7.92, *p* < 0.001; N: *F* = 4.18, *p* < 0.005). Soil RNA/DNA ratios did not differ significantly with site or warming (pseudo-*F* = 0.56, *p* > 0.05) (Table S2). 

**Figure 2:**
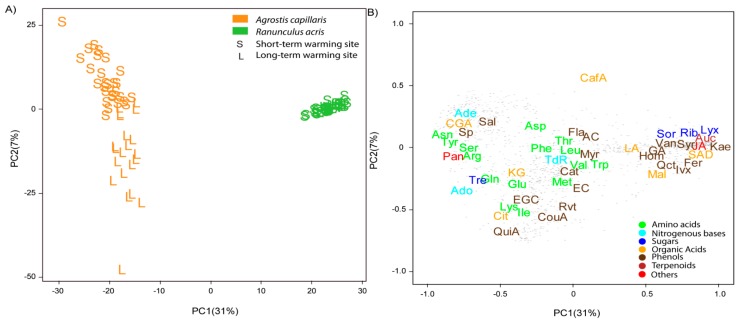
Plots of cases and variables in the principal component analysis (PCA) conducted with the physicochemical traits, elemental compositions, and biological and metabolomic variables of *Ranunculus acris* and *Agrostis capillaris* using PC1 versus PC2. (**A**) The cases are categorised by site and species. Species are indicated by different colours (green, *R. acris*; orange, *A. capillaris*). The two sites are indicated by S for short-term warming and L for long-term warming. (**B**) Loadings of the metabolomic variables in PC1 and PC2. The various metabolomic families are represented by colours: dark blue, sugars; green, amino acids; orange, compounds related to the metabolism of amino acids and sugars; cyan, nucleotides; brown, phenolics; dark red, terpenes; and red, others. Metabolites: arginine (Arg), asparagine (Asn), aspartic acid (Asp), glutamic acid (Glu), glutamine (Gln), isoleucine (Ile), lysine (Lys), leucine (Leu), methionine (Met), phenylalanine (Phe), serine (Ser), tryptophan (Trp), threonine (Thr), tyrosine (Tyr), valine (Val), adenine (Ade), adenosine (Ado), thymidine (TdR), chlorogenic acid (CGA), trans-caffeic acid (CafA), α-ketoglutaric acid (KG), citric acid (Cit), L-malic acid (Mal), lactic acid (LA), succinic acid (SAD), pantothenic acid hemicalcium salt (Pan), jasmonic acid (JA), 5,7-dihydroxy-3,4,5-trimethoxyflavone (Fla), acacetin (AC), epicatechin (EC), epigallocatechin (EGC), homoorientin (Hom), isovitexin (Ivx), kaempferol (Kae), myricetin (Myr), quercetin (Qct), resveratrol (Rvt), saponarin (Sp), catechin hydrate (Cat), 3-coumaric acid (CouA), gallic acid (GA), quinic acid (QuiA), sodium salicylate (Sal), syringic acid (Syr), trans-ferulic acid (Fer), vanillic acid (Van), 2-deoxy-D-ribose (Rib), D-(−)-lyxose (Lyx), D-(+)-sorbose (Sor), D-(+)-trehalose dehydrate (Tre), aucubin (Auc). Unassigned metabolites are represented by small grey points.

A principal component analysis (PCA) found that PC1 accounted for the differences in the metabolomes between species, whereas PC2 separated the samples of both sites (Figure 2), consistent with the results of the PERMANOVA. PCs 1 and 2 (including species, site, and warming) explained 31% and 7% of the variance, respectively. Species was the primary factor, and site was the secondary factor in explaining the differences in foliar metabolomes. The concentrations of amino acids, some compounds related to amino acids and saccharides (RCAAS), some nitrogenous bases and phenolic acids, and most organic acids, were higher in *A. capillaris* than *R. acris* leaves (Figure 2). The concentrations of some saccharides, such as ribose, lyxose, sorbose, and trehalose, organic acids such as malic acid, and some phenolic acids, were higher in *R. acris* than *A. capillaris* leaves.

### 3.2. Effects of Length and Level of Warming on the Metabolome of A. capillaris

PCs 1 and 2 explained 13% and 8% of the variance, respectively, in the PCA conducted with the foliar samples (including site and warming) of *A. capillaris* plants (Figure 2). PC1 accounted for the differences between sites. Foliar RNA/DNA ratios were higher at the site of long-term warming than the site of short-term warming.

**Figure 3:**
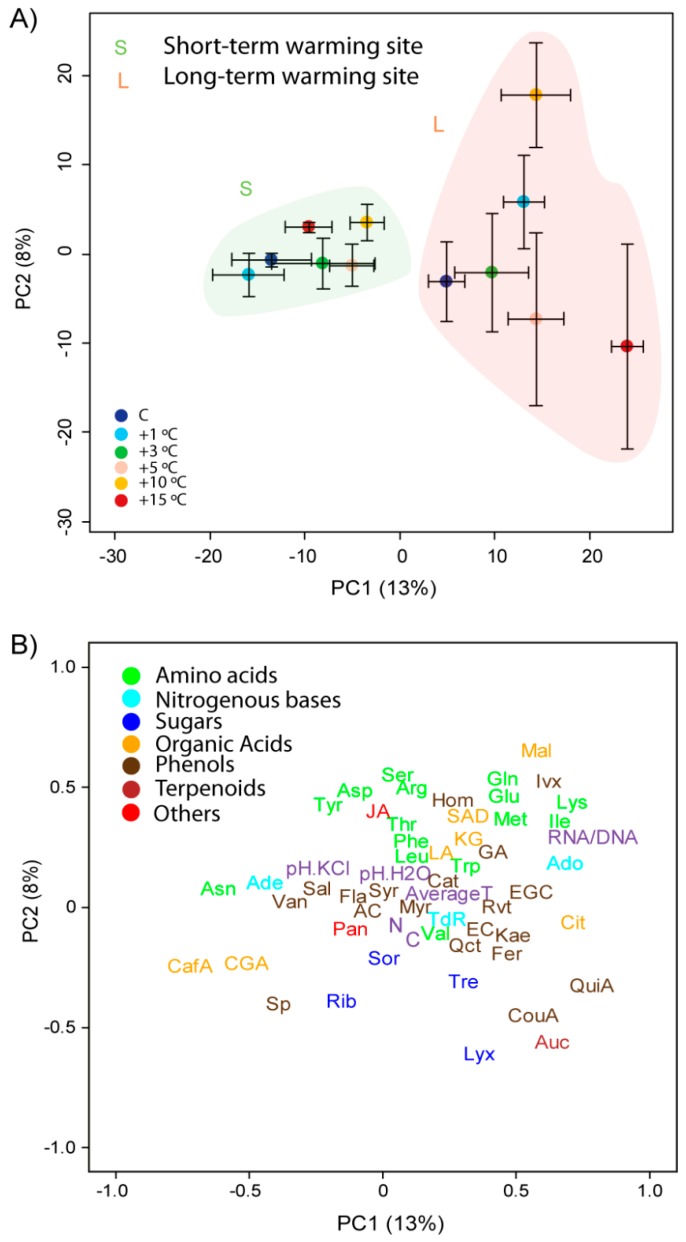
Plots of cases and variables in the PCA conducted with the physicochemical traits, elemental compositions, and biological and metabolomic variables of *Agrostis capillaris* using PC1 versus PC2. (**A**) The samples are categorised by scores (mean ± S.E.) for both sites (S for short-term warming and L for long-term warming). (**B**) Loadings of the various physicochemical, biological, and metabolomic variables in PC1 and PC2. Physicochemical variables, C and N concentrations, and the RNA/DNA ratio are shown in purple. The various metabolomic families are represented by colours: dark blue, sugars; green, amino acids; orange, compounds related to the metabolism of amino acids and saccharides; cyan, nucleotides; brown, phenolic acids; dark red, terpenes; and red, others. Metabolites as in Figure 2. Unassigned metabolites as in Figure 2 are not depicted in this figure.

**Figure 4:**
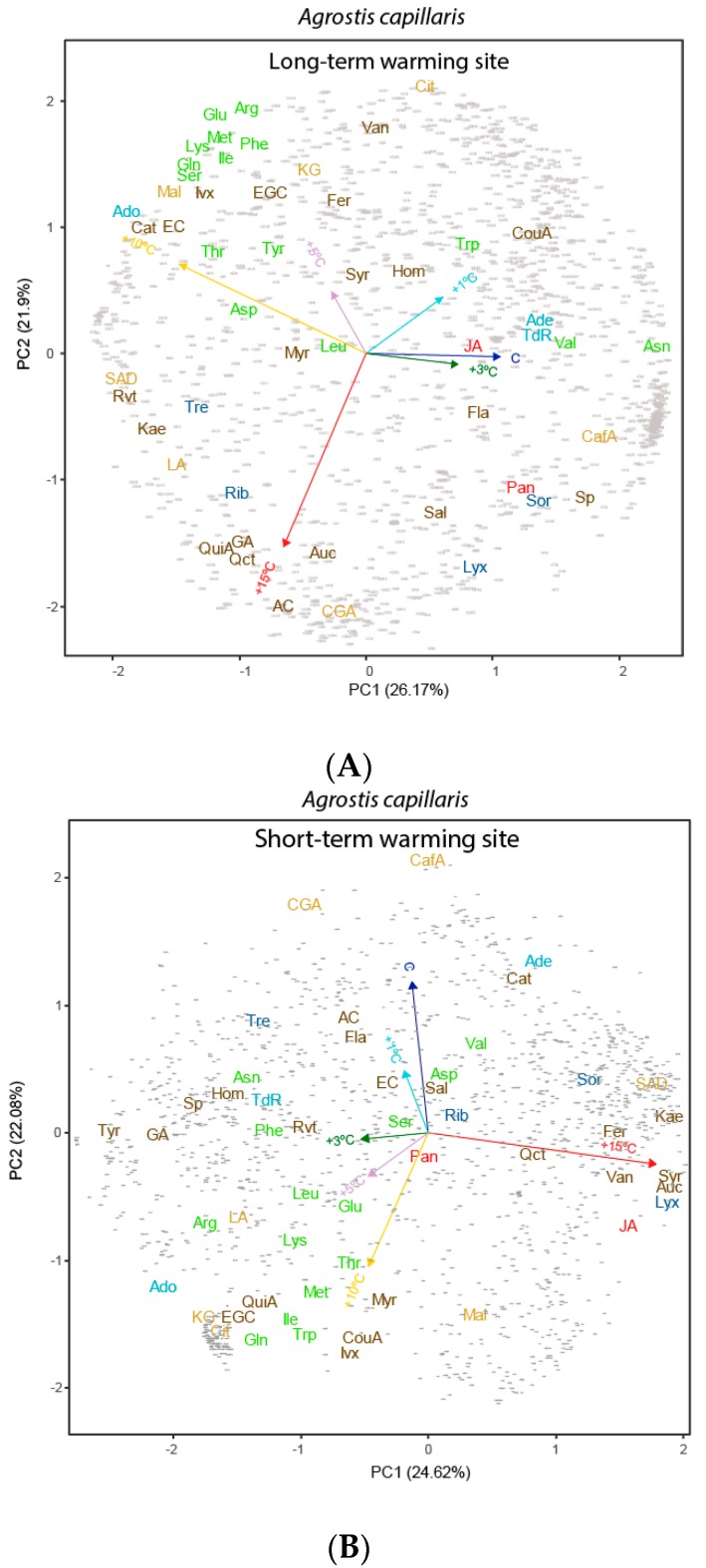
Biplots of the PC1–PC2 plane resulting from the ANOVA-simultaneous component analysis (ASCA) conducted for the six warming treatments for *Agrostis capillaris* in each site: (**A**) in Long-term warming site and (**B**) in Short-term warming site. The arrows indicate the five temperature variables. The data included the 1640 detected metabolites including the unknown compounds represented by grey points. The various metabolomic families are represented by colours: dark blue, sugars; green, amino acids; orange, compounds related to the metabolism of amino acids and sugars; cyan, nucleotides; brown, phenolics; dark red, terpenes; and red, others. Metabolites: arginine (Arg), asparagine (Asn), aspartic acid (Asp), glutamic acid (Glu), glutamine (Gln), isoleucine (Ile), lysine (Lys), leucine (Leu), methionine (Met), phenylalanine (Phe), serine (Ser), tryptophan (Trp), threonine (Thr), tyrosine (Tyr), valine (Val), adenine (Ade), adenosine (Ado), thymidine (TdR), chlorogenic acid (CGA), trans-caffeic acid (CafA), α-ketoglutaric acid (KG), citric acid (Cit), L-malic acid (Mal), lactic acid (LA), succinic acid (SAD), pantothenic acid hemicalcium salt (Pan), jasmonic acid (JA), 5,7-dihydroxy-3,4,5-trimethoxyflavone (Fla), acacetin (AC), epicatechin (EC), epigallocatechin (EGC), homoorientin (Hom), isovitexin (Ivx), kaempferol (Kae), myricetin (Myr), quercetin (Qct), resveratrol (Rvt), saponarin (Sp), catechin hydrate (Cat), 3-coumaric acid (CouA), gallic acid (GA), quinic acid (QuiA), sodium salicylate (Sal), syringic acid (Syr), trans-ferulic acid (Fer), vanillic acid (Van), 2-deoxy-D-ribose (Rib), D-(−)-lyxose (Lyx), D-(+)-sorbose (Sor), D-(+)-trehalose dehydrate (Tre), aucubin (Auc).

**Figure 5:**
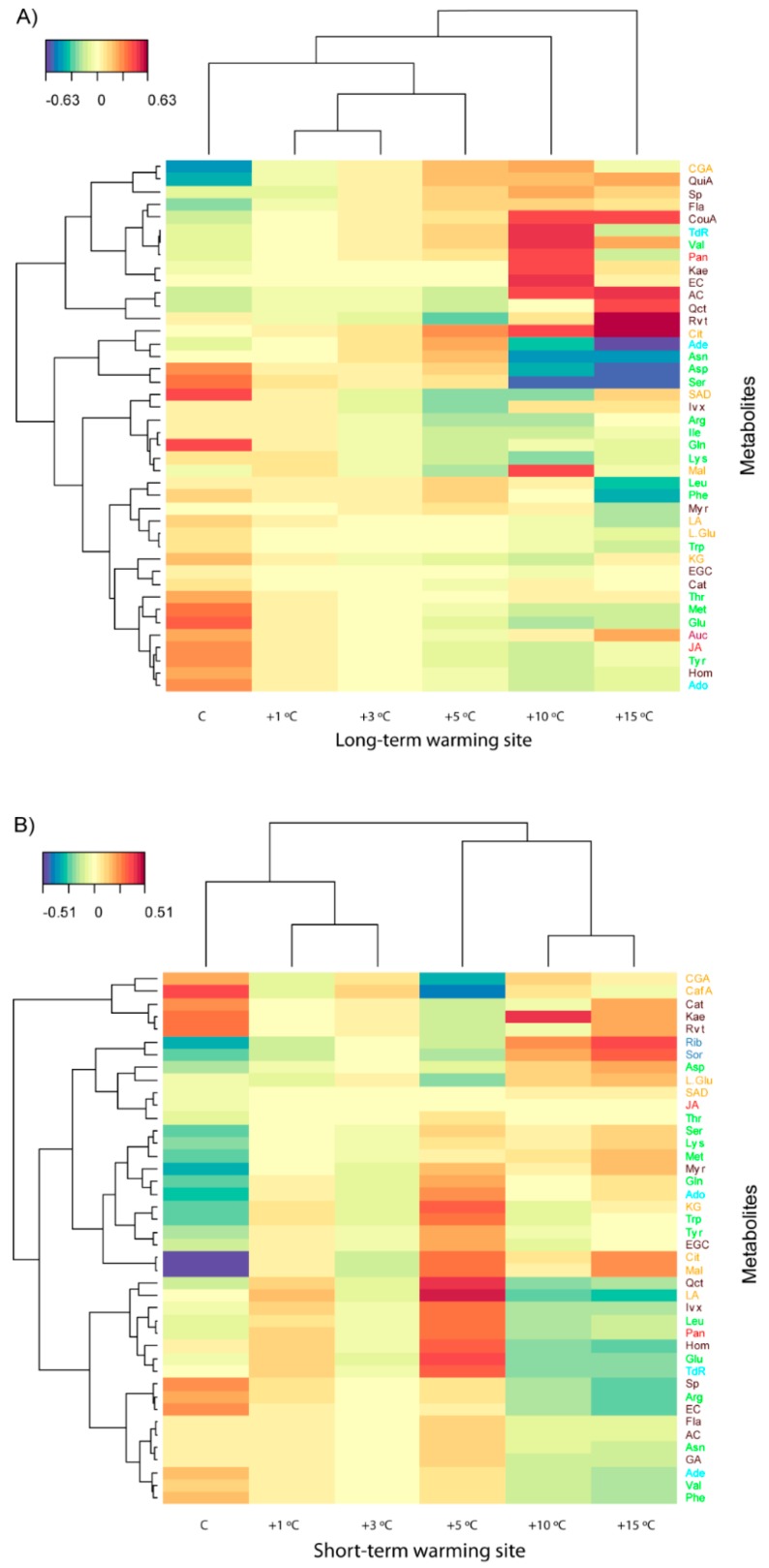
(**A**) Clustered image maps of the metabolites of *Agrostis capillaris* at the sites of (**A**) long-term warming and (**B**) short-term warming based on the data of the PLS analysis. Red and blue indicate positive and negative correlations, respectively (for more details Supplementary Information).

The PCA identified differences amongst the warming levels, which was supported by the PERMANOVA; *A. capillaris* metabolomes at higher and lower temperatures differed substantially at the LW site, but less at the SW site (Figure 3 and Table 1 and Table 2). Extreme warming conditions (+15 °C) induced the accumulation of most phenolic acids and terpenes in *A. capillaris* leaves at the LW site. High warming conditions (+10 °C), however, led to the accumulation of some amino acids and malic acid in the leaves (Figure 3, Figure 4 and Figure 5).

The PCA did not identify differences amongst the physicochemical variables, such as soil pH, or C or N concentrations, between sites. Soil pH and temperature were correlated with metabolomic shifts at both sites, and the foliar RNA/DNA ratio was higher at the long-term site.

### 3.3. Effects of Length and Level of Warming on the Metabolome of R. acris

**Figure 6:**
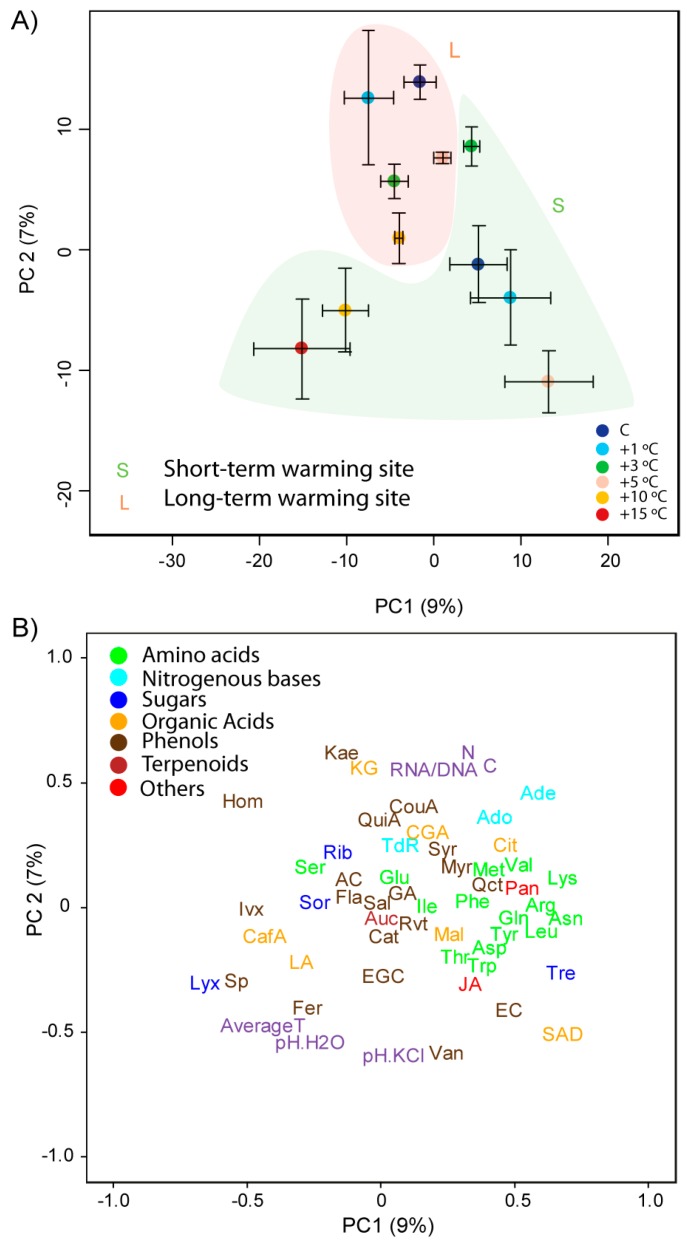
Plots of cases and variables in the PCA conducted with the physicochemical traits, elemental compositions, and biological and metabolomic variables of *R. acris* using PC1 versus PC2. (**A**) The samples are categorised by scores (mean ± S.E.) at both sites (S for short-term warming and L for long-term warming). (**B**) Loadings of the various physicochemical, biological, and metabolomic variables in PC1 and PC2. Physicochemical variables, C and N concentrations, and the RNA/DNA ratio are shown in purple. The various metabolomic families are represented by colours: dark blue, saccharides; green, amino acids; orange, compounds related to the metabolism of amino acids and sugars; cyan, nucleotides; brown, phenolic acids; dark red, terpenes; and red, others. Metabolites as in Figure 2. Unassigned metabolites, as in Figure 2, are not depicted in this figure.

**Figure 7:**
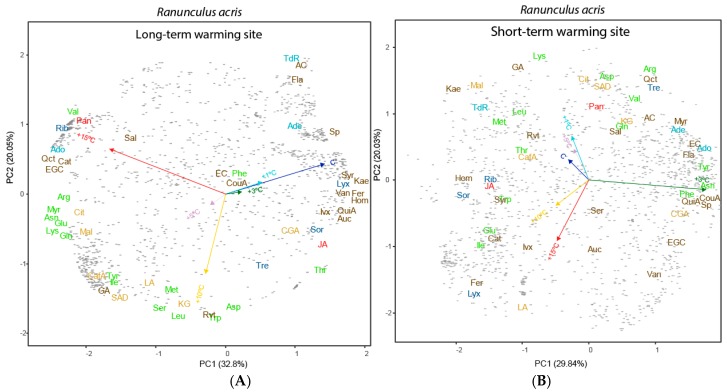
Biplots of the PC1–PC2 plane resulting from the ANOVA-simultaneous component analysis (ASCA) conducted for the six warming treatments for *Ranunculus acris* in each site. (**A**) In Long-term warming site and (**B**) in Short-term warming site. The arrows indicate the five temperature variables. The data included the 1640 detected metabolites including the unknown compounds represented by grey points. The various metabolomic families are represented by colours: dark blue, sugars; green, amino acids; orange, compounds related to the metabolism of amino acids and sugars; cyan, nucleotides; brown, phenolics; dark red, terpenes; and red, others. Metabolites: arginine (Arg), asparagine (Asn), aspartic acid (Asp), glutamic acid (Glu), glutamine (Gln), isoleucine (Ile), lysine (Lys), leucine (Leu), methionine (Met), phenylalanine (Phe), serine (Ser), tryptophan (Trp), threonine (Thr), tyrosine (Tyr), valine (Val), adenine (Ade), adenosine (Ado), thymidine (TdR), chlorogenic acid (CGA), trans-caffeic acid (CafA), α-ketoglutaric acid (KG), citric acid (Cit), L-malic acid (Mal), lactic acid (LA), succinic acid (SAD), pantothenic acid hemicalcium salt (Pan), jasmonic acid (JA), 5,7-dihydroxy-3,4,5-trimethoxyflavone (Fla), acacetin (AC), epicatechin (EC), epigallocatechin (EGC), homoorientin (Hom), isovitexin (Ivx), kaempferol (Kae), myricetin (Myr), quercetin (Qct), resveratrol (Rvt), saponarin (Sp), catechin hydrate (Cat), 3-coumaric acid (CouA), gallic acid (GA), quinic acid (QuiA), sodium salicylate (Sal), syringic acid (Syr), trans-ferulic acid (Fer), vanillic acid (Van), 2-deoxy-D-ribose (Rib), D-(−)-lyxose (Lyx), D-(+)-sorbose (Sor), D-(+)-trehalose dehydrate (Tre), aucubin (Auc).

PC2 accounted for the differences in the metabolomes of *R. acris* at the sites of short- and long-term warming (Figure 5). PCs 1 and 2 explained 9% and 7% of the variance, respectively, in the PCA conducted with the foliar samples (including site and warming). The metabolomes were more distinct amongst plants submitted to different soil temperatures at the SW than the LW site (Figure 4). The metabolomes differed between the two sites and the levels of warming. The metabolomes differed substantially at the highest (+10 and +15 °C) and moderate warming levels (Table 3) at the short-term site but did not differ with soil temperature at the long-term site (Figure 6 and Figure 7 and Table 3 and Table 4).

Various physicochemical variables in the PCA, such as soil pH and stoichiometry, were distinctly correlated with the metabolomic shifts at the two sites. Soil pH and temperature were correlated with metabolomic differences in *R. acris* at the SW site, and the foliar RNA/DNA ratio and soil C and N concentrations were higher at the LW site.

**Figure 8:**
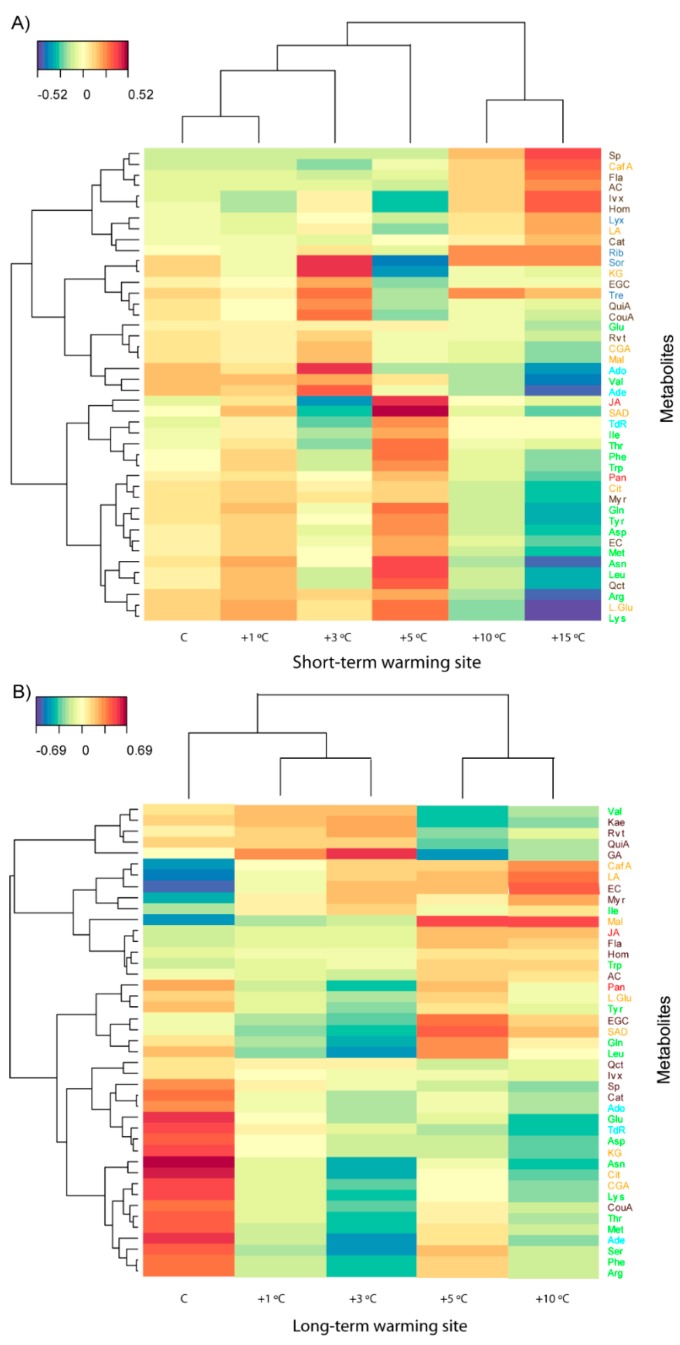
Clustered image maps of the metabolites of *Ranunculus acris* at the sites of (**A**) short-term warming and (**B**) long-term warming based on the data of the PLS analysis. Red and blue indicate positive and negative correlations, respectively (for more details, refer to supplementary information).

Increases in phenolic acids, such as coumaric acid, quinic acid, saponarin, and resveratrol, some terpenes, and saccharides, were detected in plants growing under the extreme warming conditions. In contrast, the concentrations of some amino acids and organic acids, such as malic acid, were higher in *R. acris* plants growing under moderate warming (Figure 6, Figure 7 and Figure 8).

The metabolomes of *R. acris* plants growing at soil temperatures of +10 and +15 °C at the short-term site were clearly separated from those in the other treatments, and the plants growing at different soil temperatures at the long-term site differed less in metabolomic structure (Figure 5). The PCA identified differences amongst the warming levels at both sites, consistent with the results of the PERMANOVA (Table 4).

Soil total C and N concentrations were negatively correlated with metabolomes associated with increasing temperature in *R. acris*, and the foliar RNA/DNA ratio was negatively correlated with metabolomes associated with increasing temperature.

## 4. Discussion

Changes in temperature strongly affected the synthesis of multiple metabolites and metabolic pathways in both studied species (Table 5). Assimilated C was allocated to growth and energy supply (primary metabolism) under moderate warming up to +5 °C, conditions favourable for growth. Assimilated C, under less favourable conditions of high warming levels, >+5 °C, however, was allocated increasingly to anti-stress compounds, such as phenolic acids and terpenes (secondary metabolism). Warming had an abrupt effect on the metabolome of both species at levels >+5 °C, changing the metabolomes significantly (Figure 4, Figure 5 and Figure 6; Table 5 and Table S1), mainly by shifting the C allocation from primary metabolism to secondary metabolism. The current scenarios of climate change project temperatures over land to rise by between +2 and +8 °C at high latitudes [1] (Figure 1 and Figure 6). The results thus strongly suggest that if the projected increase surpasses +5 °C, both species, but especially *A. capillaris*, may respond to this increase in temperature with a down-regulation of primary metabolism and an up-regulation of secondary metabolism. The translation of these responses to soil warming, to responses to the higher atmospheric temperatures, however, needs caution. In a global warming scenario, the high temperatures should affect aboveground plant organs directly, whereas in warming coming from soil, the impacts are firstly and strongly on roots, that thereafter, would affect aboveground plant organs. Notably, in the studied sub-arctic area, only two clear seasons exist: summer, when the temperature allows the existence of liquid water in soil surface and thus biological activity; and winter, when soil is frozen and very low plant activity exists. Thus, the study provides the metabolomics snapshot under different soil temperatures in the biologically most active part of the year.

Metabolomic variability was lower in *R. acris* than *A. capillaris* (Figure 2). The differences in the metabolomes of individuals growing at different soil temperatures were much smaller for *R. acris* than *A. capillaris*. The coefficients of variation of the PC2 scores were 15% for *R. acris*, and 57% for *A. capillaris*. These results strongly suggest that metabolism is much more conservative and homeostatic for *R. acris* than *A. capillaris*, consistent with the higher metabolomic homeostasis and lower flexibility to environmental changes in herbaceous than grass species previously reported in other ecometabolomic studies [63,64]. The metabolome of the grass species did not change significantly with short-term soil warming, but did after long-term warming, where it had more time to adapt. This trend was not observed in *R. acris*. 

The RNA/DNA ratio for *A. capillaris* was also higher at the highest warming level in the LW site, but not in the SW site. This suggested that transcriptomic activity or gene expression and protein synthesis were higher in the populations at the LW site, than the populations at the SW site. This was consistent with the shift in metabolomic profile at higher temperatures in the populations observed in the LW site, but not clearly observed in the SW site. A selection towards more metabolically active individuals with long-term warming is thus suggested in *A. capillaris*. Warmed *A. capillaris* plants in LW site had more active metabolic pathways coupled with the biosynthesis of saccharides and amino acids, but also with some important groups of secondary metabolites, such as phenolic acids and terpenes. This includes higher concentrations of amino acids and their derivatives (citric acid, threonine, glutamine, glutamic acid, and malate), phenolic acids, and terpenes with increasing soil warming. These results are consistent with previous findings of similar metabolomic shifts under drought or warming conditions towards the biosynthesis of soluble saccharides and amino acids involved in anti-heat stress mechanisms [65]. Among the observed metabolites that increased their concentrations in *A. capillaris* under higher levels of soil warming at the LW site, several have been associated to heat stress responses: proline, glycine betaine, and soluble sugars and amino acids (Diamant et al., 2001; Wahid, 2007), sugar alcohols (polyols), tertiary and quaternary ammonium compounds (Sairam and Tyagi, 2004), terpenes such as aucubin [66], and phenolics and derivates (Maestri et al., 2002; Bita and Gerats, 2013). In fact, higher phenolics, soluble saccharides, and amino acid concentrations in plant tissues have been directly associated with anti-thermal stress mechanisms, by increasing cell membranes and complex protein clusters’ stability (Kaplan et al., 2004; Rizhisky et al., 2004; Coucheney et al., 2008; Michaud et al., 2008; Puskal et al., 2010; Ancilloti et al., 2015; Sung et al., 2003; Mirzaei et al., 2012), to increases in cellular osmotic potential (Ancillotti et al., 2015; Fumagalli et al., 2009; Puskal et al., 2010; Rizhsky et al., 2004), and in the case of phenolics, to antioxidant effects (Maestri et al., 2002; Bita and Gerats, 2013). The high concentration of secondary metabolites is usually a result of complex regulatory processes associated with the production of reactive oxygen species [67]. Moreover, an extreme increase in soil temperature can lead to water deficits, and thereby, the production of more soluble saccharides and amino acids that could act as osmolytes, and contribute to the maintenance of turgor by osmotic adjustment [41,68,69]. 

*A. capillaris* plants at the SW site had lower amounts of free amino acids, such as methionine, lysine, and isoleucine, at higher soil warming levels. Organic acids, saccharides, and phenolic acids accumulated at the SW site with increasing soil warming (Figure 4). This decrease in free amino acids with warming, could be associated with the incorporation of amino acids into heat-stress proteins (Bray et al., 2000). The amount of asparagine, a metabolite that plays a role in translocation and storage of nitrogen and contributes to the maintenance of osmotic pressure, however, increased with warming [70]. The metabolome of *A. capillaris* at the SW site also had higher levels of jasmonic acid at higher warming levels. Jasmonic acid acts as a regulator of plant growth and development and is converted to a variety of derivatives, including their esters, that may also be conjugated to amino acids [71]. Metabolomes of A. capillaris were thus clearly different in SW and LW sites. In the SW site, the amino acids increased with moderate warming, but in the LW site they decreased. Moreover, in LW site, >+5 °C, the concentrations of most determined amino acids increased, and several secondary compounds also increased. These increases were, by contrast, not observed in SW site at >+5 °C.

*R. acris* in the warming treatments of the SW sites >+5 °C, had higher amounts of metabolites, such as sucrose and glucose, that are involved in the response to heat shock, and are usually associated with higher concentrations of amino acids [35]. It did not have larger concentrations of amino acids (asparagine, leucine, isoleucine, threonine, alanine, and valine), but of derivatives, such as oxaloacetate and pyruvate [41], that are associated with higher concentrations of sugars. Tyrosine, found in high amounts in warming treatments of *R. acris*, has an important role in photosynthesis, where it acts as an electron donor in the reduction of oxidised chlorophylls. Tyrosine may also influence the amount of stress hormones [72], and is a precursor of some alkaloids [73]. Phenylalanine, accumulated at the highest levels of soil warming, is the substrate of phenylalanine ammonia lyase, a key enzyme in phenolic biosynthesis, consistent with the increase in phenolic compounds at the highest warming levels. These phenolic acids are thought to protect plants against abiotic and biotic stresses [74]. The metabolome of *R. acris* at the LW site under moderate warming (from control to +5 °C) contained higher amounts of polyphenolics, such as quinic acid, and organic acids (Figure 3 and Figure 4). Organic acids such as malic acid, chlorogenic acid, quercetin, and α-ketoglutaric acid, are associated with the Krebs cycle. α-Ketoglutaric acid is an intermediate of the Krebs cycle, and a precursor of glutamine and glutamate biosynthesis. These compounds have been frequently associated with antioxidant function. Quinic acid is a precursor in the shikimic acid pathway, a common metabolic pathway in the biosynthesis of aromatic amino acids, such as tyrosine, tryptophan, and phenylalanine [75]. These amino acids, in turn, are precursors of a large variety of secondary metabolites such as lignins, flavonoids, alkaloids, and phytodexins [76]. *R. acris* at the long-term site under high warming levels (+5 to +10 °C) had higher amounts of phenolic acids, terpenes, and flavonols, such as kaempferol and quercetin, that could have a protective effect against biotic stressors [74,75].

On average, *R. acris* had higher amounts of free amino acids, most phenolic and organic acids, and saccharides, at the SW than the LW site (Figure 4 and Figure 6). The amounts of nitrogenous bases and flavonols, and the foliar RNA/DNA ratio, however, were higher at the LW site. We should expect that this perennial herb, with a long generation time and with general traits closer to stress-tolerant species, had less metabolomic flexibility than the grass species, *A. capillaris*. Consistently with this, the metabolomic variability amongst individual *R. acris* plants growing at different temperatures tended to be low over time in both sites. The greater capacity of seed dispersal of *R. acris* should promote the mixing of genotypes from different locations (with different soil temperatures) at both sites, which could account for this lower variability amongst sites with different temperatures. The amounts of free amino acids and some nucleotide bases in *R. acris* plants were higher at the lower, than at higher, warming levels at both the SW and LW sites. Thus, differently than in *A. capillaris*, the warming effects on metabolome structure were similar in SW, than in LW sites, in *R. acris*. The stimulation of biosynthesis of amino acids indicates an activation of primary metabolism. In both sites, there seems to be a downregulation of primary metabolism at the higher soil temperatures related to the decrease of the concentration of amino acids and nucleotide bases. Moreover, *R. acris* had higher amounts of organic acids, such as lactic acid and trans-caffeic acid; sugars, such as lyxose, ribose, sorbose, and trehalose; and secondary metabolites, such as phenols at the SW than at the LW site (Figure 5 and Figure S2). These patterns were in accordance with a previous study reporting an accumulation of non-reducing disaccharides, such as lyxose, during periods of stress [76].

## 5. Conclusions

Different responses were detected in the two plant species, depending on the length of time the plant communities had been exposed to warming. The metabolomic composition of *A. capillaris* changed more at the site of long-term than short-term warming. The metabolome of *R. acris* was less responsive to soil warming.

Plants at the long-term warming site contained higher amounts of saccharides and amino acids. This up-regulation of primary metabolism coincided with a higher foliar RNA/DNA ratio. The plants at the long-term site also accumulated some secondary metabolites, such as phenolic acids and terpenes that can have an important protective effect against both heat-related and biotic stressors. 

A thermal threshold, indicated by an abrupt shift and nonlinear response in overall metabolomic profiles, was observed between +5 and +10 °C soil warming for both *A. capillaris* and *R. acris*. Above this threshold, both species tended to up-regulate the metabolic pathways associated with heat stress.

## Figures and Tables

**Table 1:** PERMANOVA for the metabolite data set, physicochemical variables, and the RNA/DNA ratio for *Agrostis capillaris*. Bold type indicates significant effects (*p* < 0.05), and italics indicate a marginal effect (*p* < 0.1).

	Df	*F*	*P*
Site	1	13.3136	**0.0005**
Temperature	5	1.6354	**0.0210**
Site × Temp	5	1.4068	*0.0815*

**Table 2:** *p*-Values of the pair-wise comparisons of post hoc Tukey’s HSD tests from the one-way ANOVA between the PC1 scores of the PCA of the metabolome of *Agrostis capillaris*. The table shows the results of *t*-test statistics for the comparisons of PCA scores for *A. capillaris* at the sites of (**A**) long-term warming and (**B**) short-term warming. Bold type indicates significant effects (*p* < 0.05), and italics indicate marginal effects (*p* < 0.1).

**(A)**					
***Agrostis capillaris* L. Long-Term Warmed Site**					
Temperature	+1 °C	+3 °C	+5 °C	+10 °C	+15 °C
Control	0.92	1.000	1.00	**0.01**	**0.047**
+1 °C		0.936	0.92	*0.08*	**0.004**
+3 °C			1.00	**0.04**	**0.01**
+5 °C				*0.05*	**0.05**
+10 °C					**0.03**
**(B)**					
***Agrostis capillaris* L. Short-Term Warmed Site**					
Temperature	+1 °C	+3 °C	+5 °C	+10 °C	+15 °C
Control	1.00	0.70	0.23	**0.02**	*0.08*
+1 °C		0.93	0.49	**0.04**	*0.07*
+3 °C			0.98	*0.07*	*0.07*
+5 °C				1.00	*0.09*
+10 °C					0.82

**Table 3:** Post hoc Tukey’s HSD tests from the one-way ANOVA. The table shows the results of *t*-test statistics for the comparisons of the PCA scores for *Ranunculus acris* for the various warming levels at the sites of (**A**) long-term warming and (**B**) short-term warming. Bold type indicates significant effects (*p* < 0.05), and italics indicate marginal effects (*p* < 0.1).

**(A)**					
***Ranunculus acris* L. Long-Term Warmed Site**					
Temperature	+1 °C	+3 °C	+5 °C	+10 °C	
Control	***0.05***	0.14	0.99	0.40	
+1 °C		0.92	***0.05***	0.53	
+3 °C			0.12	0.91	
+5 °C				0.31	
**(B)**					
***Ranunculus acris* L. Short-Term Warmed Site**					
Temperature	+1 °C	+3 °C	+5 °C	+10 °C	+15 °C
Control	0.99	1.00	0.74	**0.02**	**0.02**
+1 °C		0.96	0.97	**0.007**	**0.006**
+3 °C			0.62	**0.03**	**0.03**
+5 °C				**0.01**	**0.001**
+10 °C					0.89

**Table 4:** PERMANOVA for the metabolite data set, physicochemical variables, and the RNA/DNA ratio for *Ranunculus acris*. Bold type indicates significant effects (*p* < 0.05).

	Df	*F*	*P*
Site	1	2.9063	**0.0005**
Temperature	5	1.9687	**0.0005**
Site × Temp	4	1.0586	0.2334

**Table 5:** Summary of the overall up- and down-regulation of the metabolomic pathways groups. The arrows indicate relative values.

Site	Long-Term Warming Site												Short-Term Warming Site											
Spe	Agrostis Capillaris						Ranunculus Acris						Agrostis Capillaris						Ranunculus Acris					
Temp	A	B	C	D	E	F	A	B	C	D	E	F	A	B	C	D	E	F	A	B	C	D	E	F
Amino acid metabolism	↑↑	↓	-	-	↑↑	↑↑	-	-	↑	↓	↓	↓	-	-	-	-	↑↑	↑↑↑	-	-	-	↓	↑↑↑	↑↑
Biosynthesis of other secondary metabolites	-	↓	-	-	↑↑↑	↑↑	-	↓↓	↑↑	↓	-	↑↑	-	-	↓↓	↑	↑	↑↑↑	↑↑	-	-	-	↑↑	-
Carbohydrate metabolism	-	-	↑	-	↑↑	-	↑	↓	↓↓	-	↓	↓↓↓	-	-	↓↓	-	-	↑	↓	↓	↓	-	↑↑↑	↑↑
Energy metabolism	-	-	↑	-	↓	-	-	-	↓↓↓	-	↓↓	↓↓↓	↑	↑↑	-	↑↑	-	-	↑	↑	↑↑	↓	↓↓	-
Metabolism of cofactors and vitamins	↑↑↑	↑↑↑	↑↑	↑↑	↑	↑↑	↓	↓	↓	-	↓↓	↓↓↓	↑	↑	-	↑↑	↑	-	-	↓	-	↓↓↓	↓↓↓	↓↓
Metabolism of other amino acids	↑↑↑	-	↑↑↑	↑↑	↑	↑↑↑	↓↓	↓	↓	↓↓↓	↓↓	↓	↑↑↑	↑↑	↑	↑↑	↑↑	↑↑	↓↓	↓↓	↓↓	↓↓↓	↓↓↓	↓↓
Metabolism of terpenoids and polyketides	↑↑	↓↓	-	↑	↑	↑↑	↓	↓↓↓	-	↓↓↓	↓↓	↓↓↓	-	↑↑	-	↑↑	-	↑↑	↑	-	↑	-	-	-
Xenobiotics biodegradation and metabolism	-	↑	↑	-	-	-	↓	↓↓↓	↓↓↓	↓↓↓	↓↓↓	↓↓↓	↓↓	↓↓	↓↓	↑	↑	-	↓↓	↓↓	↓↓	↑↑	↑↑↑	↑↑↑

## Supplementary Materials

The following are available online at www.mdpi.com/2218-1989/7/3/44/s1, Figure S1: Cross validation of the PCA, Table S1: Post hoc Bonferroni tests from the one-way ANOVA, Table S2: One-way ANOVAs of the physicochemical and biological traits of the soils, Table S3: Processing parameters of the LC-MS chromatograms using MZmine 2.10.
