# Supplementary material for: Impact of Soil Warming on the Plant Metabolome of Icelandic Grasslands
Source: Metabolites. 2017 Aug 23;7(3):44. doi: 10.3390/metabo7030044 (PMC5618329; doi:10.3390/metabo7030044)
Supplement: Supplementary file 1 [file metabolites-07-00044-s001.pdf]

Supporting information: Impact of Soil Warming on the Plant Metabolome of Icelandic Grasslands

Albert Gargallo-Garriga [1,2,*], Marta Ayala-Roque [1,2,*], Jordi Sardans [1,2], Mireia Bartrons [1,3], Victor Granda [1,2], Bjarni D. Sigurdsson [4], Niki I. W. Leblans [4,5], Michal Oravec [6], Otmar Urban [6], Ivan A. Janssens [5] and Josep Peñuelas [1,2]

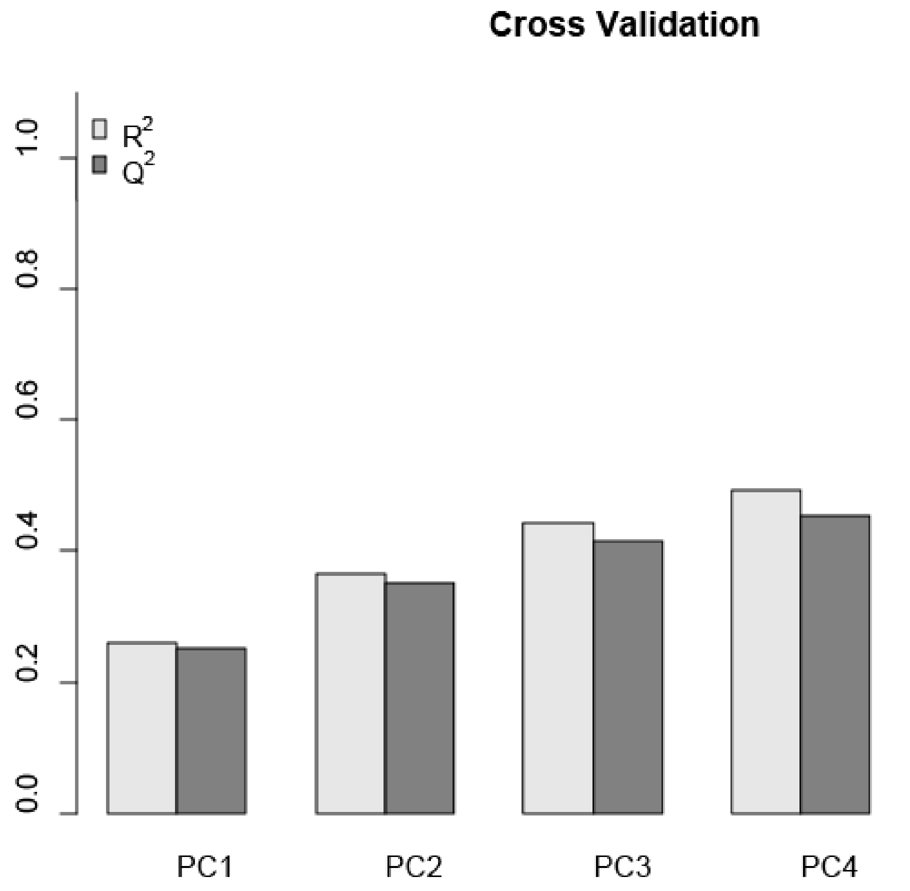

Figure S1: Cross validation of the PCA.

Table S1. Post hoc Bonferroni tests from the one-way ANOVA. The table shows the results of *t*-test statistics for the comparisons of PCA scores for the different warming levels for both species, *Agrostis capillaris* and *Ranunculus acris*. Bold type indicates significant effects ($P < 0.05$), and italics indicate marginal effects ($P < 0.$ 1).

Temperature	+1 °C	+3 °C	+5 °C	+10 °C	+15 °C
Control	0.79	0.13	0.96	**<0.001**	**0.001**
+1 °C		0.80	0.99	**0.01**	*0.05*
+3 °C			0.60	**0.02**	*0.05*
+5 °C				**0.005**	**0.03**
+10 °C					**0.005**

Tables S2. One-way ANOVAs of the physicochemical and biological traits of the soils. Bold type indicates significant effects (P < 0.05), and Italics indicate a marginally significant effect (P < 0.1).

	**Df**	**Average T**		**pH H_2O**		**pH KCl**		**RNA:DNA**		**%C**		**%N**	
		F	*P*	*F*	*P*	*F*	*P*	*F*	*P*	*F*	*P*	*F*	*P*
Site	1	8.70	**0.01**	5.42	**0.03**	10.8	**0.001**	0.56	0.46	10.5	**0.001**	155	**0.001**
Temp	5	127	**0.001**	6.03	**0.001**	2.14	*0.09*	0.98	0.44	7.92	**0.001**	4.18	**0.005**
Site*Temp	5	1.52	0.21	0.46	0.80	0.41	0.84	0.87	0.51	1.61	0.18	3.10	**0.02**

Table S3. Processing parameters of the LC-MS chromatograms using MZmine 2.10 [47]. The chromatograms correspond to the total ion current (TIC).

	**(+H) Chromatograms**	**(-H) Chromatograms**
**Baseline correction**		
Chromatogram type	TIC	TIC
MS level	1	1
Smoothing	10E6	10E6
Asymmetry	0.001	0.001
**Mass detection** (Exact mass)		
Noise level	4.5×10^5	4.5×10^5
**Chromatogram builder**		
Min time span	0.05	0.05
Min height	25000	25000
m/z tolerance	0.002	0.002
**Smoothing**		
Filter width	5	5
**Chromatogram deconvolution (Local minimum search)**		
Chromatographic threshold	70%	70%
Search minimum in RT range (min)	0.1	0.1
Minimum relative height	7.0%	7.0%
Minimum absolute height	30000	30000
Min ratio of peak top/edge	2	2
Peak duration range	0.0–2.0	0.0–2.0
**Chromatogram alignment (join alignment)**		
m/z tolerance	0.001	0.001
Weight for m/z	80	80
RT tolerance	0.3	0.3
Weight for RT	20	20
**Gap filling (Peak Finder)**		
Intensity tolerance	20%	20%
m/z tolerance	0.001	0.001
Retention time tolerance	0.1	0.1
RT correction	marked	marked
**Filtering**		
Minimum peaks in a row	25	25
**Ions excluded from database**	<75<br>Between 0.0 and 1 min<br>Between 28.5 and 30 min	<85<br>Between 0.0 and 1.1 min<br>Between 27.0 and 30 min

CIM Supplementary information

CIM or heatmaps were introduced in (Eisen et al., 1998) to represent data resulting from gene expression profiles. This type of representation is based on a hierarchical clustering simultaneously operating on the rows and columns of a real-valued similarity matrix M. The initial matrix is graphically represented as a 2-dimensional colored image, where each entry of the matrix is colored on the basis of its value, and where the rows and columns are reordered according to a hierarchical clustering. Dendrograms resulting of the clustering are added to the left (or right) side and to the top (or bottom) of the image. With sPLS we chose to display CIM based on the pair-wise similarity matrix defined. These approaches introduce l1 (Lasso) penalization terms on the loading vectors to shrink some of the coefficients towards zero, thus allowing for simultaneous variables selection in the two data sets. The pair-wise similarity matrix was then computed using our proposed method (see Section 'Methods') for the first three PLS dimensions in order to display the CIM. The Euclidian distance and the Ward method were used for the hierarchical clustering. In the CIM display, each colored block represents an association between subsets of the X-variables and the Y-variables. The red color indicates that the X and Y clusters are positively correlated, and the red color indicates a negative correlation in the X-Y cluster, whereas blue indicate weaker correlation values. The dendrograms on the top and the left hand side of the map indicate how the clusters join, the longer the distance, the sharper the boundary between the colored blocks. The variables with blank names indicate variables with weak correlations (irrelevant variables). The CIM details the correlations between all variables in a more comprehensive manner than the correlation circle plots. Following the referee's advice, we have now added this information to the revised version in the supplementary information.
